# Healthy Diet Intervention for Treating Atopic Dermatitis: A Pilot Randomised Controlled Trial

**DOI:** 10.1111/cea.70282

**Published:** 2026-03-13

**Authors:** Jun Jie Lim, Hafizah Yusri, Dimeng Yang, Jung Eun Kim, Mei Hui Liu, Fook Tim Chew

**Affiliations:** ^1^ Department of Biological Sciences, Faculty of Science National University of Singapore Singapore; ^2^ Department of Food Science & Technology, Faculty of Science National University of Singapore Singapore

**Keywords:** atopic dermatitis, clinical trial, dermatology, diet, nutrition, skin barrier

## Abstract

- Short‐term SCORAD improvement observed with structured dietary intervention; long‐term between‐arm effects require cautious interpretation.
- Larger, longer trials are needed to confirm efficacy and durability.

To the Editor,

Atopic dermatitis (AD) is a chronic inflammatory skin disease that frequently persists into adulthood and substantially impairs quality of life, sleep, and psychosocial well‐being [1]. While pharmacological therapies remain central to management, incomplete responses and concerns regarding long‐term use have prompted interest in complementary, non‐pharmacological strategies [2]. Dietary modification has emerged as a potentially modifiable adjunct; however, adult intervention trials remain limited and have predominantly focused on single‐nutrient supplementation or elimination‐based approaches rather than whole‐diet modification [3, 4, 5]. Evidence from Asian adult populations is particularly scarce [5].

Building on our prior epidemiological findings demonstrating that frequent intake of saturated fat (SFA)‐rich foods was associated with higher odds of AD exacerbation, whereas greater consumption of fruits, vegetables and dietary fibre was associated with lower odds [6, 7, 8], we conducted a pilot, parallel‐arm, assessor‐blinded randomised controlled trial (RCT). The RCT evaluated whether a culturally adapted healthy dietary pattern (HDP), aligned with Singapore's My Healthy Plate (MHP) guidelines [9], could reduce AD severity as measured by clinical symptoms.

Adults aged 21–65 years with physician‐diagnosed AD were recruited in Singapore and randomised 1:1 to either a HDP intervention or a habitual‐diet control. Diagnosis was confirmed at screening by trained dermatology personnel using established clinical criteria based on the Hanifin and Rajka framework. To preserve ethical equipoise, control participants were not instructed to modify or increase fat intake but were asked to maintain their habitual diet, reflecting real‐world baseline dietary behaviour. Participants with severe disease requiring systemic therapy, major comorbidities, restricted diets (including vegetarian or vegan diets), or recent adherence to HDPs were excluded to ensure participant safety and interpretability of dietary effects in this pilot study. Outcome assessors and data analysts were blinded to arm allocation, and participants were instructed not to discuss dietary allocation during assessments.

Intervention participants received daily lunch and dinner aligned with MHP guidelines for 8 weeks, supported by structured counseling to promote adherence. Control participants continued usual care without dietary counselling. The primary outcome was change in Scoring Atopic Dermatitis (SCORAD). Secondary outcomes included skin physiology, Dermatology Life Quality Index (DLQI), medication use, anthropometry, and lipid‐lipoprotein profiles. The trial was registered (NCT06547372), approved by the National University of Singapore Institutional Review Board (NUS‐IRB‐2024‐28), and all participants provided written informed consent.

Fifty‐seven participants completed the study (diet: *n* = 28; control: *n* = 29) (Figure 1A). Baseline characteristics were comparable between arms, with most participants presenting moderate AD. In the diet arm, mean SCORAD (± standard error of the mean) declined from baseline (43.4 ± 1.3) to week 8 post‐intervention (29.0 ± 1.8) and showed short‐term persistence at week 12 follow‐up (28.2 ± 1.9), meeting minimal clinically important difference thresholds. Between‐arm differences in SCORAD change were statistically significant at all post‐baseline time points (all *p* < 0.001; Figure 1B).

**FIGURE 1:**
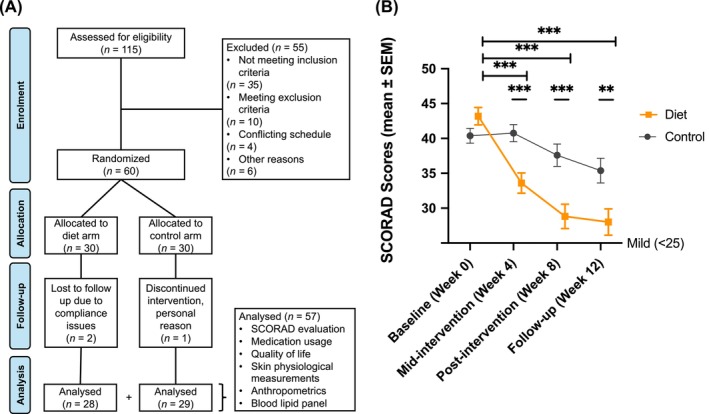
Participant recruitment and primary outcome. (A) Flowchart of participant recruitment, allocation, and follow‐up. A total of 57 participants completed the study and were included in the final analysis (diet arm: *n* = 28, control arm: *n* = 29). (B) Mean SCORAD ± standard error of the mean (SEM) over time in the diet and control arms. A SCORAD score < 25 indicates mild atopic dermatitis. Single connecting lines with asterisks denote statistically significant between‐arm differences at each post‐baseline time point. Bracketed lines denote within‐arm changes from baseline. Statistical significance is indicated as *p < 0.05, **p < 0.01, and ***p < 0.001. Study time points are week 0 (baseline), week 4 (mid‐intervention), week 8 (post‐intervention), and week 12 (follow‐up).

Exploratory analyses of skin physiology showed modest within‐arm changes in the diet arm; however, no statistically significant between‐arm differences were observed for transepidermal water loss or skin hydration, and these findings should be interpreted cautiously. Concomitant medication use was systematically recorded throughout the study and remained low and stable over time, with no differential trends between arms. No significant between‐arm differences were observed for DLQI, medication use, anthropometry or lipid–lipoprotein profiles.

Dietary intake was assessed longitudinally using repeated 3‐day food diaries at baseline, during the intervention, and post‐intervention (weeks 9 and 11). Healthy Eating Index (HEI) was used to assess overall dietary pattern adherence rather than individual nutrients, while quantitative estimates of SFA intake were evaluated separately during the intervention period. During the intervention, participants in the diet arm demonstrated higher adherence to a HDP and lower estimated SFA intake compared with controls. Following cessation of meal provision, HEI scores declined toward habitual patterns, underscoring the short‐term nature of dietary adherence in the absence of ongoing support. Importantly, the intervention was well tolerated over the study period, with no safety signals identified beyond passive adverse event monitoring.

This pilot RCT has several limitations. The intervention and follow‐up duration preclude conclusions regarding long‐term sustainability. Participant blinding was not inherently feasible, and given the psychosomatic sensitivity of AD, expectancy or placebo effects may have influenced subjective outcomes. Although SCORAD includes both objective and subjective components and was assessor‐administered, we acknowledge that inclusion of the Eczema Area and Severity Index, a HOME‐recommended core outcome instrument, could have provided a fully objective, blinded assessment of AD severity and further minimised potential bias related to participant non‐blinding. Medication use was captured using frequency categories rather than quantitative dose or potency, and differences in application technique or intensity cannot be fully excluded; however, the absence of between‐arm differences in medication use reduces the likelihood that observed clinical changes were driven primarily by pharmacologic escalation. The intervention combined dietary modification with behavioural support; thus, observed effects reflect a composite dietary intervention and should be interpreted as a proof‐of‐concept rather than diet‐specific causality. The selective study population and exclusion criteria inevitably constrain generalizability to older adults, severe AD, individuals with comorbidities, or resource‐limited settings. Finally, mechanistic pathways were not directly measured, and the proposed biological explanation associated with clinical improvements remains hypothesis‐generating.

Despite these limitations, this pilot RCT demonstrates the feasibility and short‐term clinical benefit of a culturally adapted, supported whole‐diet intervention delivered as an adjunct to standard dermatological care. Larger, longer‐duration trials (≥ 6 months), incorporating crossover designs or attention‐matched comparators, extended follow‐up, and mechanistic assessments are required to determine durability, isolate diet‐specific effects and evaluate real‐world scalability.

## Author Contributions

The authors' responsibilities were as follows: F.T.C. conceived and supervised the study and secured funding. F.T.C., J.E.K., M.H.L. and J.J.L. contributed to the conceptualisation and design of the study. J.J.L., H.Y. and D.Y. conducted participant recruitment and data collection. J.J.L. performed the data analysis and literature review. Interpretation of findings was undertaken by J.J.L., J.E.K., M.H.L., and F.T.C. J.J.L. drafted the original manuscript. J.E.K. and M.H.L. provided methodological expertise. J.J.L. and J.E.K. critically reviewed the manuscript. All authors approved the final manuscript.

## Funding

FTC received grants from the National University of Singapore (N‐154‐000‐038‐001 (E‐154‐00‐0017‐01); C141‐000‐077‐001 (E‐141‐00‐0096‐01)), Singapore Ministry of Education Academic Research Fund (R‐154‐000‐191‐112; R‐154‐000‐404‐112; R‐154‐000‐553‐112; R‐154‐000‐565‐112; R‐154‐000‐630‐112; R‐154‐000‐A08‐592; R‐154‐000‐A27‐597; R‐154‐000‐A91‐592; R‐154‐000‐A95‐592; R‐154‐000‐B99‐114), Biomedical Research Council (BMRC) (Singapore) (BMRC/01/1/21/18/077; BMRC/04/1/21/19/315; BMRC/APG2013/108), Singapore Immunology Network (SIgN‐06‐006; SIgN‐08‐020), National Medical Research Council (NMRC) (Singapore) (NMRC/1150/2008; OFIRG20nov‐0033; MOH‐001636 (OFLCG23may‐0038, A‐8002641‐00‐00)), National Research Foundation (NRF) (Singapore) (NRF‐MP‐2020‐0004), Singapore Food Agency (SFA) (SFS_RND_SUFP_001_04; W22W3D0006; NRF‐SFSRND2SIH‐0001; SFS_RND_2_FS_0002), Singapore's Economic Development Board (EDB) (A‐8002576‐00‐00), and the Agency for Science Technology and Research (A*STAR) (Singapore) (H17/01/a0/008; and APG2013/108). This research is supported by the National Research Foundation Singapore under its Open Fund‐Large Collaborative Grant (MOH‐001636) (A‐8002641‐00‐00) and administered by the Singapore Ministry of Health's National Medical Research Council. The funding agencies had no role in the study design, data collection and analysis, decision to publish, or preparation of the manuscript.

## Ethics Statement

The study was approved by the National University of Singapore Institutional Review Board (NUS‐IRB‐2024‐28) and registered at clinicaltrials.gov (NCT06547372).

## Consent

All participants provided written informed consent, and the study was conducted in accordance with the Declaration of Helsinki.

## Conflicts of Interest

F.T.C. reports grants from the National University of Singapore, Singapore Ministry of Education Academic Research Fund, Singapore Immunology Network, National Medical Research Council (NMRC) (Singapore), Biomedical Research Council (BMRC) (Singapore), National Research Foundation (NRF) (Singapore), Singapore Food Agency (SFA), Singapore's Economic Development Board (EDB), and the Agency for Science Technology and Research (A*STAR) (Singapore), during the conduct of the study; and consulting fees from Sime Darby Technology Centre; First Resources Ltd., Genting Plantation, Olam International, Musim Mas, and Syngenta Crop Protection, outside the submitted work. The other authors declare no other competing interests. This research is supported by the National Research Foundation Singapore under its Open Fund‐Large Collaborative Grant (MOH‐001636) (A‐8002641‐00‐00) and administered by the Singapore Ministry of Health's National Medical Research Council. None of the authors has a conflicts of interest to declare.

## Data Availability

The data that support the findings of this study are openly available in Dietary Intervention on Atopy at https://osf.io/6w7k3/overview?view_only=a049dbe674b84da8a0981a9bdd33bbf1. Further data that support the findings of this study are available upon reasonable request from the corresponding author (FTC).
